# Targeting ALK in Neuroendocrine Tumors of the Lung

**DOI:** 10.3389/fonc.2022.911294

**Published:** 2022-06-07

**Authors:** Dilara Akhoundova, Martina Haberecker, Ralph Fritsch, Sylvia Höller, Michael K. Kiessling, Markus Rechsteiner, Jan H. Rüschoff, Alessandra Curioni-Fontecedro

**Affiliations:** ^1^Department of Medical Oncology and Hematology, University Hospital Zurich, Zurich, Switzerland; ^2^Department of Medical Oncology, Inselspital, University Hospital of Bern, Bern, Switzerland; ^3^Department for BioMedical Research, University of Bern, Bern, Switzerland; ^4^Department of Pathology and Molecular Pathology, University Hospital Zurich, Zurich, Switzerland; ^5^Institute of Pathology, Stadtspital Zurich Triemli, Zurich, Switzerland; ^6^Department of Internal Medicine–Oncology, See Spital Horgen, Horgen, Switzerland

**Keywords:** *ALK* rearrangements, neuroendocrine tumors of the lung, large cell neuroendocrine carcinoma, ALK inhibitors, targeted treatment

## Abstract

**Background:**

*Anaplastic lymphoma kinase* (*ALK*) rearrangements are known oncogenic drivers in non-small cell lung cancer (NSCLC). Few case reports described the occurrence of such rearrangements in large cell neuroendocrine carcinomas (LCNECs) of the lung without information on clinical responses to ALK tyrosine kinase inhibitors (TKIs) in these cases. Currently, neuroendocrine tumors of the lungs are not screened for *ALK* rearrangements.

**Methods:**

To illustrate the clinical impact of molecular characterization in LCNECs, we report the disease course in three patients with *ALK*-rearranged metastatic LCNEC from our clinical routine, as well as their treatment response to ALK TKIs (index cases). To gain insight into the prevalence of *ALK* rearrangements in neuroendocrine tumors of the lung, we analyzed a retrospective cohort of 436 tumor biopsies including LCNEC (n = 61), small cell lung cancer (SCLC) (n = 206), typical (n = 91) and atypical (n = 69) carcinoids, and mixed histology (n = 9) for the presence of *ALK* rearrangements using a sequential diagnostic algorithm. ALK immunohistochemistry (IHC) was evaluable in 362 cases; fluorescence *in situ* hybridization (FISH) was evaluable in 28 out of the 35 IHC-positive cases, followed by next-generation sequencing (NGS) that was available in 12 cases.

**Results:**

Within the retrospective cohort, ALK IHC was positive in 35 out of 362 (9.7%) evaluable samples. FISH was positive in 3 out of the 28 (10.7%) evaluable cases: 2 with atypical carcinoids and 1 with LCNEC. Additionally, the 3 index cases showed positive ALK IHC, which was confirmed by NGS. Within the retrospective cohort, NGS confirmed the presence of an *ALK* genomic rearrangement in one FISH-positive atypical carcinoid where material was sufficient for sequencing. Two out of three patients with metastatic *ALK*-rearranged LCNEC received up-front treatment with the ALK TKI alectinib and showed rapid tumor response at all metastatic sites, including multiple brain metastases.

**Conclusions:**

*ALK* rearrangements represent rare but targetable oncogenic driver alterations in LCNEC. Contrarily to NSCLC, the detection of *ALK* rearrangements in neuroendocrine tumors of the lung is challenging, since ALK IHC can lead to false-positive results and therefore needs confirmation by FISH or NGS. Up-front comprehensive molecular profiling with NGS should be performed in metastatic LCNEC in order not to miss actionable genomic alterations.

## Introduction

Large cell neuroendocrine carcinomas (LCNECs) constitute a rare subtype of pulmonary malignancies, accounting for approximately 3% of all primary lung cancers (LCs) (1). LCNECs usually course aggressively and exhibit a very poor prognosis in advanced disease stages (2–4). Molecular profiling of LCNEC shows profound genomic heterogeneity, harboring common features to small cell lung cancer (SCLC) and to non-small cell lung cancer (NSCLC). Frequent genomic alterations concern *TP53*, *RB1*, *STK11*, *KEAP1*, and *KRAS* (5–7). Genomic and transcriptomic analyses of a cohort of 75 LCNECs suggested the subclassification into two major molecular subtypes: type I LCNEC (37%), characterized by biallelic *TP53* and *STK11* or *KEAP1* alterations and neuroendocrine phenotype, and type II LCNEC (42%), harboring biallelic *TP53* and *RB1* inactivation. Type II LCNECs, contrary to type I, show reduced levels of neuroendocrine markers and enrichment of immune signaling pathways (8, 9).

While the majority of LCNECs develop *de novo*, more rarely, histological transformation from a distinct subtype of NSCLC has been reported (10–12). This histological transformation can occur as an acquired resistance mechanism to targeted treatment with epidermal growth factor receptor (EGFR) or anaplastic lymphoma kinase (ALK) tyrosine kinase inhibitors (TKIs) (13–17). However, the most frequent histologic transformation as resistance mechanism to targeted therapies is into SCLC (12).

Due to LCNEC genomic heterogeneity, efforts have been done to identify molecular predictors of therapeutic vulnerability. For instance, the presence of wild-type *RB1* has been associated with better survival outcome and better response to gemcitabine or taxane-containing chemotherapy regimens as compared to platin–etoposide combinations. On the other hand, no differences in chemotherapeutic sensitivities were detected for tumors harboring *RB1* loss (6). In a single-center retrospective review by Zhou et al. (18) , histopathological and clinical features from 126 patients with central (n = 45) and peripheral (n = 81) LCNEC were analyzed. In 9 out of 81 peripheral tumors (18.8%), *EGFR* alterations could be detected. One additional tumor (2.1%) harbored a *ROS1* fusion. No central LCNEC showed molecular alterations in *EGFR* or *ROS1*, and no *ALK* or *BRAF* alterations were reported. Patients with central tumors had a significantly worse survival outcome, further illustrating the biological and clinical heterogeneity of LCNEC (18).

*ALK* rearrangements drive 5%–6% of NSCLCs (19, 20). For LCNEC, very few data regarding the presence of ALK rearrangements are reported in the literature (21). Most larger molecular profiling studies in LCNEC, with 65 or 35 cases, respectively, did not detect any ALK rearrangements using comprehensive molecular profiling, including mutational, fusion, and copy number analysis (7, 22).

Additionally, ALK fusions also have been rarely found in other neuroendocrine tumors of the lung, such as typical (TCs) and atypical pulmonary carcinoids (ACs) (21), as well as in SCLC (23).

In this study, we investigated the prevalence of *ALK* alterations in a large cohort of neuroendocrine tumors of the lung, including LCNEC, SCLC, TC, and AC (n = 439) by immunohistochemistry (IHC), fluorescence *in situ* hybridization (FISH), and next-generation sequencing (NGS). Additionally, we report three clinical cases from our institution, which illustrate the clinical impact of up-front comprehensive profiling of pulmonary LCNEC.

## Materials and Methods

### Patient Cohort

Four tissue microarrays (TMAs) were constructed employing formalin-fixed paraffin-embedded (FFPE) tissue samples comprising SCLC, LCNEC, TC, AC, and mixed histology. The TMA cohort consisted of tumor samples from 436 patients with neuroendocrine lung tumors biopsied or resected between 1991 and 2012. Two cores for each patient have been included. All samples were retrieved from the Department of Pathology and Molecular Pathology, University Hospital Zurich, Switzerland. We further included tumor samples from three additional patients to this study, with identified metastatic ALK-positive LCNEC (index cases). In these cases, the negative smoking history led the clinician to perform molecular characterization as for NSCLC. Our study was approved by the local ethics committee (KEK ZH-Nr. 29-2009) and was conducted in accordance with local laws and regulations.

### ALK Immunohistochemistry

**Figure 1:**
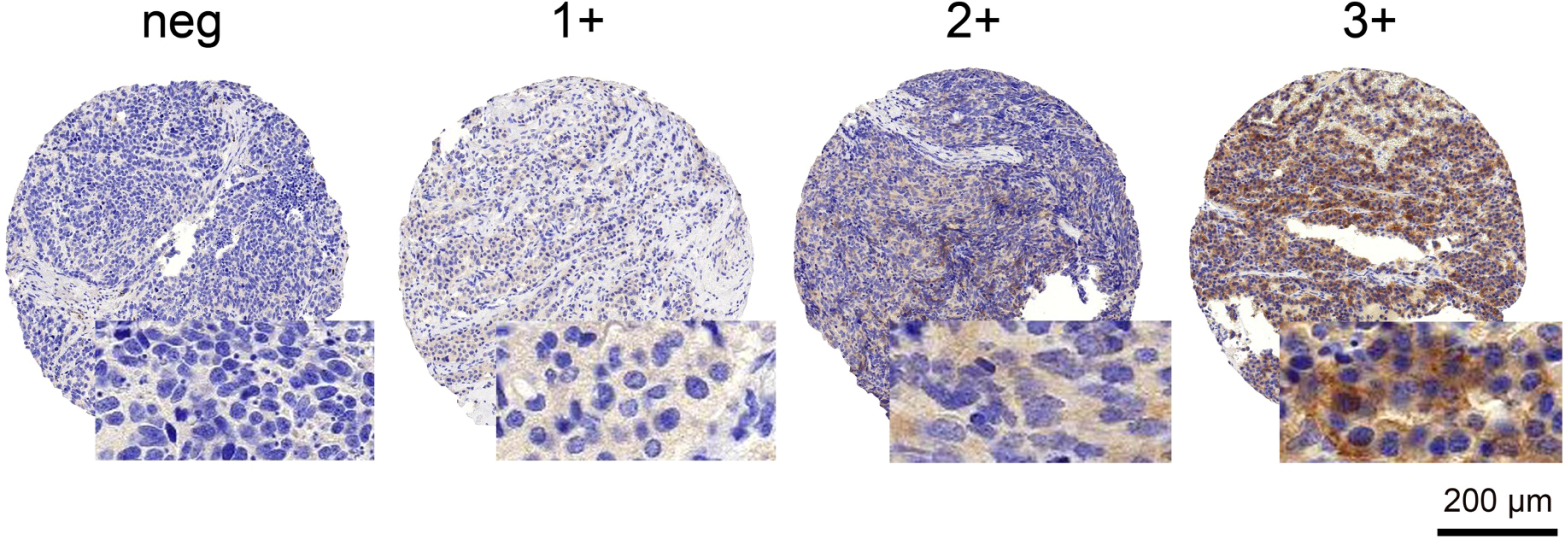
ALK IHC staining patterns. Examples of negative and positive (1+, 2+, and 3+) staining patterns are illustrated. Scale bar 200 µm. neg, negative.

ALK IHC on 2-µm-thick TMA sections was performed using a mouse monoclonal antibody for ALK (Clone 5A4; Leica, Wetzlar, Germany) at 1:10 dilution. A cell line with confirmed ALK translocation served as a positive control. ALK expression was evaluated by two experienced pulmonary pathologists (JR, MH) in a blinded manner. In case of a discrepancy in IHC scoring, a consensus score was established. The cytoplasmic staining intensity was semiquantitatively scored as follows: 0 (negative), 1+ (weak), 2+ (moderate), or 3+ (strong). Cases with any positivity (scores 1+, 2+, and 3+) were considered positive (**Figure 1**).

### ALK Fluorescence *In Situ* Hybridization

FISH for the detection of ALK rearrangements was performed using a commercially available ALK assay (Vysis LSI ALK Dual Colour, Break-Apart Rearrangement Probe; Abbott Molecular, IL, USA). FISH analyses were performed on all IHC-positive samples (scores 1+, 2+, and 3+) according to the manufacturer’s recommendations (24). Signals were counted in at least 50 tumor nuclei per case using an epifluorescence microscope (Zeiss, Oberkochen, Germany). FISH for ALK locus rearrangement was considered positive if at least 15% of analyzed cells showed either a split of one set of red and green signals greater than two signal widths apart and/or if loss of one green signal (5′ probe) had occurred as per Abbott Molecular scoring criteria (24–26).

### Next-Generation Sequencing

NGS was performed on the 2+ and 3+ IHC-positive cases, where enough tumor material was available. In total, 15 samples were sequenced using Oncomine™ Focus Assay (OFA) Panel (RNA-part), Archer^®^ FusionPlex^®^ Sarcoma kit or FoundationOne^®^CDx (Foundation Medicine, Cambridge, MA, USA) assay.

For OFA sequencing, RNA was isolated using Maxwell 16 LEV RNA FFPE Purification Kit (Promega). The RNA concentration was measured with Qubit. For the OFA panel, enrichment and chip loading were done on the Ion Chef with the Ion 540 Kit. The S5 platform was used for sequencing with the Ion S5 Sequencing Kit. The protocols from Life Technologies/Thermo Fisher Scientific were followed in all steps. Alignment, variant calling, and annotation were done with the Ion Reporter software 5.10 (Oncomine™ Focus v2.4-Fusions-Single Sample).

Sequencing using the Archer^®^ FusionPlex^®^ Sarcoma panel was performed using the NextSeq550 platform and the NextSeq550 High Output Kit v3 chemistry. The protocols from Archer were followed in all steps. Alignment, variant calling, and annotation were done with the Archer software Version 6.2.1.

Isolated DNA was analyzed by the hybrid capture-based NGS platform (FoundationOne^®^CDx) at the Department of Pathology and Molecular Pathology, University Hospital Zurich, Switzerland. The methods of F1CDx have been previously described (27). The current F1CDx gene panel (https://www.foundationmedicine.com) analyzes 324 genes, including *ALK* rearrangements. Additionally, microsatellite instability (MSI) and tumor mutational burden (TMB) are assessed.

## Results

### Patient Cohort

Clinicopathological characteristics of the analyzed cohort are illustrated in **Table 1**.

**Table 1:** Clinicopathological characteristics of the global cohort [retrospective cases (n = 436) and patient index cases (n = 3)].

Characteristic	N = 439
Age, median (range) (ys)	62 (18.0–88.0)
Histological subtype Typical carcinoid Atypical carcinoid LCNEC * Index cases* * Retrospective cases* SCLC Mixed histology	91 (20.7) 69 (15.8) 63 (14.6) *3* *61* 206 (46.9) 9 (2.0)
Sex, n (%) Women Men Unknown	142 (32.3) 157 (35.8) 140 (31.9)
TNM stage, n (%) I II IIIA IIIB IV * Index cases* * Retrospective cases* Unknown	104 (23.7) 29 (6.6) 31 (7.1) 4 (0.9) 14 (3.2) *3* *11* 257 (58.5)

LCNEC, large cell neuroendocrine carcinoma; n, number of samples; SCLC, small cell lung cancer; ys, years. TNM staging was performed at diagnosis. All 3 index cases were LCNEC with an initial TNM stage IV.

### ALK Immunohistochemistry

Within the retrospective cohort, a positive cytoplasmatic ALK expression (1+, 2+, and 3+) was observed in 35 out of the evaluable 362 (9.7%) cases. These cases comprised 10 TCs, 13 ACs, three LCNECs, and nine SCLCs. No samples with a mixed histology showed a positive IHC staining (**Table 2**). Not all cores within the TMA were suitable for immunohistochemical analysis, as some cores had missing tumor tissue (~15%). The three index cases had a positive ALK IHC (3+).

**Table 2:** Clinicopathological data and ALK results for all ALK IHC-positive (1+ to 3+) cases.

No.	Tumor Type	IHC score	FISH	NGS	TNM/Stage****
1 ^*^	LCNEC	3	Positive	EML4-ALK	cT1 cN3 cM1c, IVB
2 ^**^	LCNEC	3	NA	EML4-ALK	cT2 cN1 pM1c, IVB
3 ^***^	LCNEC	3	Positive	EML4-ALK	cT3 cN3 cM1a, IVA
4	AC	3	Positive	STRN-ALK	pT2 pN2 cM0, IIIA
5	LCNEC	2	Positive	NA	pT1 pN3, cM0, IIIB
6	AC	1	Positive	NA	pT2 pN2, cM0, IIIA
7	LCNEC	3	Negative	No fusion	*-*
8	SCLC	3	Negative	No fusion	*-*
9	SCLC	3	NA	No fusion	*-*
10	SCLC	3	NA	NA	–
11	SCLC	3	Negative	NA	-
12	AC	3	Negative	No fusion	*-*
13	AC	3	Negative	No fusion	*-*
14	AC	3	Negative	NA	-
15	AC	3	NA	NA	-
16	TC	3	NA	NA	-
17	TC	3	Negative	*No fusion*	*-*
18	SCLC	2	Negative	No fusion	*-*
19	AC	2	Negative	No fusion	*-*
20	AC	2	Negative	No fusion	*-*
21	TC	2	Negative	No fusion	*-*
22	TC	2	Negative	No fusion	*-*
23	TC	2	Negative	NA	-
24	LCNEC	1	Negative	NA	-
25	SCLC	1	NA	NA	-
26	SCLC	1	NA	NA	-
27	SCLC	1	Negative	NA	-
28	SCLC	1	Negative	AN	-
29	AC	1	Negative	NA	-
30	AC	1	NA	NA	-
31	AC	1	Negative	NA	-
32	AC	1	Negative	NA	-
33	AC	1	Negative	NA	–
34	TC	1	Negative	NA	–
35	TC	1	Negative	NA	–
36	TC	1	Negative	NA	–
37	TC	1	Negative	NA	–
38	TC	1	Negative	NA	–

AC, atypical carcinoid; IHC, immunohistochemistry; FISH, fluorescence in situ hybridization; LCNEC, large cell neuroendocrine carcinoma; NA, not available; NGS, next-generation sequencing; SCLC, small cell lung cancer; TC, typical carcinoid. *case report 1, **case report 2, ***case report 3, ****TNM stage at diagnosis. Bold cases: ALK rearrangement positive by FISH or NGS.

### ALK Fluorescence *In Situ* Hybridization

**Figure 2:**
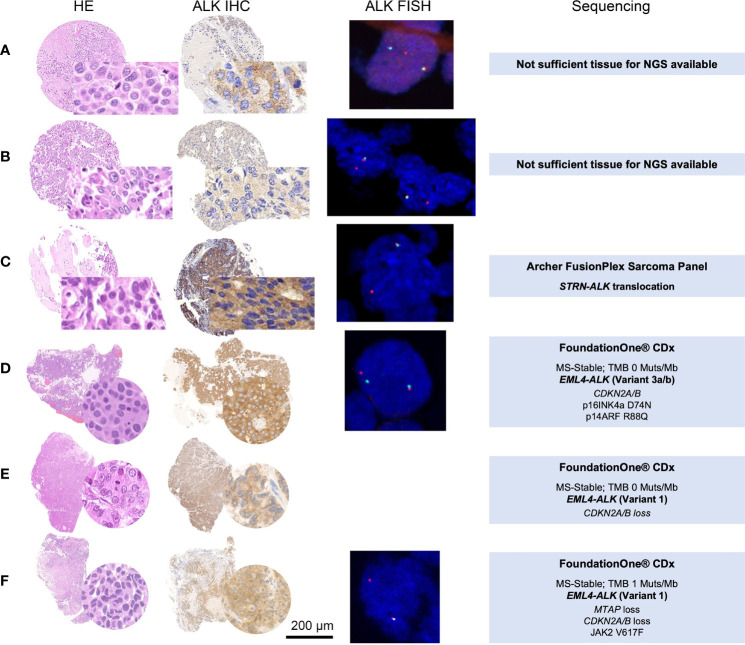
HE, ALK IHC, *ALK* FISH, and NGS sequencing data of the five *ALK* FISH-positive cases. Panels **(A**, **D**, **E**, **F)** were diagnosed as LCNEC, whereas **(B**, **C)** were evaluated as (AC). IHC was scored as 2+ **(A)**, 1+ **(B)**, and 3+ **(C–F)**. *ALK* FISH break-apart probes revealed aberrant staining patterns **(A**, **D)** one fused and one split red and green signal; **(B**, **D**, **E)** one fused and one single red signal) consistent with *ALK* rearrangements. In three cases **(C–F)**, the material was sufficient to perform NGS, confirming two times an *EML4-ALK* translocation **(D)** Variant 3a/b; **(E**, **F)** Variant 1) and one time an *STRN-ALK* translocation **(C)**. HE, hematoxylin–eosin; ALK, anaplastic lymphoma kinase; IHC, immunohistochemistry; FISH, fluorescence *in situ* hybridization; NGS, next-generation sequencing. Scale bar 200 µm.

In three out of 28 (10.7%) evaluable cases from the retrospective cohort, an *ALK* rearrangement detected by FISH was observed. The positive cases showed either a split of one set of red and green signals or loss of one green signal (5′ probe). The positive cases consisted of two AC and one LCNEC (**Figure 2** and **Table 2**).

### Next-Generation Sequencing

Within the retrospective cohort, 12 out of the 35 cases with 2+ and 3+ ALK staining by IHC had enough material to perform an NGS. Only 1 AC out of the 12 sequenced cases showed an *ALK* rearrangement. This case demonstrated a rare *STRN-ALK* fusion (breakpoint: chr2:37143221, chr2:29446394). Of the three LCNEC index cases, two harbored the typical *EML4*-*ALK* rearrangement, variant 1 (breakpoint chr2:42522656,chr2:29446394) and variant 3a/b (breakpoint: chr2:42492091,chr2:29446394*).* The three LCNEC cases showed low TMB (0, 0, and 1) and were microsatellite stable (**Table 2**).

### Clinical and Histological Characteristics of ALK-Positive Tumors

The three *EML4*-ALK-rearranged cases corresponded to biopsies from patients with LCNEC stage IV, never smokers, and with brain metastatic disease (**Table 2**). Disease course data and response to ALK TKIs are exposed under *Clinical Case Reports*. The fourth positive case by NGS, showing a rare *STRN-ALK* fusion, was an archive case and did not receive targeted treatment, therefore was not included as a clinical case report.

## Clinical Case Reports

### Case 1

A 37-year-old male patient was referred to our department with advanced LCNEC, presenting with multiple (more than 50) brain metastases, cT1 cN3 cM1c, The Union for International Cancer Control (UICC) Stage IVB, according to TNM Staging System 8th Edition (2017). The patient had never smoked. Due to symptomatic bleeding of one of the largest brain metastases, primary resection was indicated. Molecular profiling performed on the resected brain metastases showed IHC 3+ positivity for *ALK*, with no further alterations in *EGFR*, *KRAS*, *BRAF*, or *ROS1*. Programmed death-ligand 1 (PD-L1) staining (Klon E1L3N) showed expression on tumor cells of <1% (TC0) and expression on immune cells of ≥1% and <5% (IC1).

**Figure 3:**
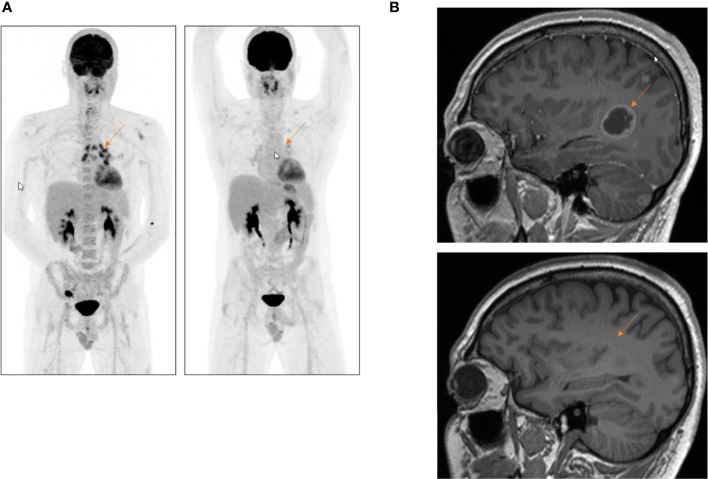
First-line treatment with alectinib in a 37-year-old patient with a metastatic *ALK*-rearranged LCNEC. **(A)** FDG-PET scan, whole-body overview, before (left) and 12 weeks after (right) initiation of first-line treatment with alectinib. **(B)** Complete response of the multiple brain metastases 10 weeks after the start of alectinib. Whole-brain radiation could be avoided. Red arrow shows excellent tumor response under treatment with alectinib.

The patient started first-line treatment with alectinib, showing good clinical and radiographical partial response of the brain metastases already 4 weeks after treatment initiation (**Figures 3A, B****)**. Since neurological symptoms rapidly improved after treatment initiation, no whole-brain radiation therapy (WBRT) was required. Under treatment with alectinib, a nearly complete response of the primary tumor, as well as of the lymph node metastasis, was observed. After 10 months on alectinib, disease progression was observed, and treatment was switched to third-generation ALK TKI lorlatinib following current clinical standards, with ongoing tumor response for already 12 months.

### Case 2

**Figure 4:**
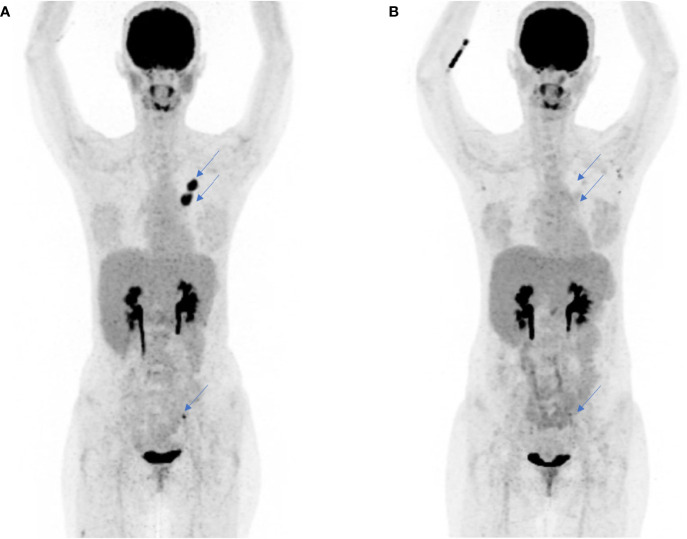
Complete metabolic and subtotal morphologic response to molecularly targeted treatment with alectinib in a 32-year-old patient diagnosed with a metastatic *ALK*-rearranged LCNEC. **(A)** FDG-PET scan, whole-body overview, before initiation of first-line treatment with alectinib. No metabolic or morphological response had been observed after one cycle of platin-based chemotherapy administered before obtaining the results of tumor molecular profiling showing *ALK* rearrangement. **(B)** FDG-PET scan performed 8 weeks after treatment start with alectinib showing complete metabolic and subtotal morphological response of the primary tumor and the lymph node metastases (blue arrows).

A 32-year-old female never smoker presented with metastatic LCNEC of the lung, with multiple small brain metastases, a single hilar lymph node metastasis, and a symptomatic metastasis to the ovary (cT2 cN1 pM1c, UICC Stage IVB, according to TNM Staging System 8th Edition, 2017). The patient underwent palliative ovariectomy and stereotactic radiosurgery of all brain metastases based on individual decision after discussion with the patient. The patient was asymptomatic regarding brain metastases at that time point. A first cycle of chemotherapy with carboplatin–etoposide was administered, while up-front NGS was pending. NGS unexpectedly showed an *EML4-ALK* rearrangement. Fluorodeoxyglucose (FDG)-PET/CT scan was performed, showing no metabolic or morphological response (stable disease) 4 weeks after the first chemotherapy application, and first-line treatment was switched to alectinib. The patient showed a rapid excellent tumor response to alectinib, with complete metabolic remission at all metastatic sites 6 weeks after treatment initiation (**Figure 4**). The patient is currently on treatment with alectinib since 5 months.

### Case 3

A 68-year-old female patient was diagnosed with LCNEC, cT3 cN3 cM1a (pleural), UICC Stage IVA. First-line chemotherapy with carboplatin and etoposide was initiated, and a very good partial response was observed after 3 cycles. After multidisciplinary discussion on thoracic tumor board, since a single pleural metastasis was initially observed and given the good tumor response, radiotherapy to the primary tumor and lymph nodes was indicated, as well as continuation of chemotherapy. The patient completed 6 cycles of chemotherapy and received 66 Gy on the primary tumor. Three months later, the patient presented with diffuse bone pain and clinical deterioration. Radiographical assessment confirmed the presence of multiple bone, hepatic, lymph node, and brain metastases. Because the patient had never smoked, a molecular profiling by NGS was performed, showing a 3+ positive staining for ALK, which was confirmed by NGS. Treatment with alectinib was initiated, but unfortunately, after a few days, the patient presented rapid clinical deterioration, ultimately succumbing to her disease.

## Discussion

LCNECs constitute a rare group of pulmonary malignancies clinically characterized by an aggressive disease course and limited benefit from conventional platin-based polychemotherapy regimens (28–30). The molecular landscape of LCNEC is heterogeneous and dominated by yet not actionable genomic alterations such as *TP53*, *RB1*, and *STK11/KEAP1* (8).

Based on the 3 illustrated *ALK*-rearranged index cases out of our clinical routine, we aimed to analyze the prevalence of *ALK* rearrangements in a retrospective cohort of 436 neuroendocrine tumors of the lung, including LCNEC, SCLC, TCs, and ACs. To our knowledge, this is one of the largest published series reviewing the prevalence of *ALK* rearrangements in neuroendocrine pulmonary tumors. In our series, 35/362 (9.7%) of all screened tumors, evaluable by IHC, showed any positive staining for ALK (score 1+, 2+, or 3+). FISH for *ALK* rearrangement was positive uniquely in three cases (1 LCNEC and 2 ACs). NGS confirmed the rearrangement in the FISH-positive AC where enough material was available for sequencing. In the global cohort (retrospective and index patients), the four positive LCNEC cases showed IHC scores of 3+, 3+, 3+, and 2+, whereas the AC cases revealed scores of 1+ and 3+. This supports previously published data from NSCLC, where ALK IHC 2+ or 3+ shows the best concordance with the presence of *ALK* genomic rearrangements (31, 32). Still, the majority of analyzed cases with ALK positivity by IHC showed no genomic rearrangements in *ALK* when performing FISH or NGS. Relevantly, all SCLCs and TCs with a 2+ or 3+ score in our cohort had no ALK rearrangement by NGS. Previous work has shown that ALK positivity by IHC is an insufficient surrogate of the presence of genomic rearrangements in SCLC and LCNEC. For example, Kondoh et al. (23) evaluated the immunohistochemical expression of ALK in 142 SCLCs, which was positive in 11.3% of the cases and all negative by FISH or real-time PCR. In another study, 27 LCNECs were analyzed, and although four cases were initially considered positive per IHC, only one genomic rearrangement was confirmed (33).

Our study shows that, unlike in NSCLC, IHC is an insufficient tool for the diagnosis of ALK rearrangement in neuroendocrine tumors of the lung due to the high prevalence of false-positive cases and low specificity. Also in other tumor entities, false-positive ALK expression has been reported, e.g., in neuroblastoma mediated by *MYCN* amplification (34). For NSCLC, on the contrary, ALK positivity by IHC is usually considered diagnostic and leads to treatment indication with ALK TKIs, despite that also some false-positive and discordant cases with FISH and NGS have been reported (35). These false-positive cases have been related to the presence of ALK amplifications or activating mutations (35, 36).

In our cohort, all three *ALK*-rearranged LCNECs harbored the classical *EML4*-*ALK* fusion and are further characterized by low TMB and microsatellite stability (**Figure 2**). The *ALK-*rearranged AC showed an *STRN-ALK* rearrangement. This is a rare fusion and has only been described in a few case reports across different tumor entities (37, 38). In NSCLC, two case reports of late-stage adenocarcinomas harboring this rearrangement exhibited an excellent response to treatment with alectinib and crizotinib, respectively (39, 40). The patient with *STRN-ALK*-rearranged AC from our cohort presented with an initial stage pT2 pN2 cM0 but developed bone and brain metastases, receiving a platin-based chemotherapy and no targeted treatment. One limitation of our study is the fact that for all IHC-positive cases, not enough tumor material was available for further confirmation of *ALK* rearrangement by either FISH or NGS.

We identified *ALK* rearrangements in our 3 index cases and one additional case out of the 61 (1.6%) retrospectively reviewed LCNEC and in 2 out of 69 (2.9%) analyzed AC cases. Clinically, the three reported index patients with *ALK*-rearranged LCNEC presented with an aggressive course of metastatic disease and multiple brain metastases. The two patients who underwent up-front molecular profiling with consequent early detection of *ALK* rearrangement showed a profound and rapid response to ALK TKIs. Additionally, due to up-front detection of *ALK* rearrangement, WBRT could be avoided in a 37-year-old patient (Case 1), who is still responding to second-line targeted treatment with lorlatinib. The illustrated index cases underline the clinical relevance of up-front screening for actionable genomic alterations in patients with advanced LCNEC. Kim et al. (41) performed comprehensive molecular characterization (NGS, whole-transcriptome sequencing, and IHC) of a cohort of 467 LCNECs, and *ALK* fusions were identified in 1.7% of the cases. In a case series, published by Zheng et al. (21) , the authors identified 4 cases of pulmonary neuroendocrine tumors (2 LCNECs, 1 AC, and 1 high-grade neuroendocrine carcinoma). One LCNEC was uniquely identified by ALK-positive IHC. An LCNEC and AC showed an *EML4-ALK* fusion, and the high-grade neuroendocrine carcinoma, a *KLC1-ALK* fusion. Interestingly, 2 out of these 4 patients, one LCNEC and one atypical metastatic carcinoid, developed brain metastases. Both tumors presenting an *EML4-ALK* fusion responded to treatment with the ALK TKI crizotinib (21). No treatment response data were available for the case harboring the *KLC1-ALK* fusion. Further case reports have shown efficacy of ALK TKIs in LCNEC with confirmed *ALK* rearrangement (42). Importantly, all 3 index *ALK*-rearranged cases were metastatic at diagnosis. On the contrary, most of the patients in the retrospective cohort had a localized disease stage at diagnosis and time point of the biopsy or tumor resection. Due to the limitation of the sample size, our study cannot answer the question whether *ALK* rearrangement occurs more frequently in metastatic LCNEC.

The updated molecular testing guidelines for the selection of lung cancer patients for treatment with targeted TKIs, reviewed in 2018 by The College of American Pathologists (CAP) and the International Association for the Study of Lung Cancer (IASLC), do not recommend the systematic molecular profiling of neuroendocrine tumors of the lung (43). Despite that targetable alterations are rare in LCNEC, our work shows the high clinical relevance of screening for *ALK* alterations in these tumors.

In summary, we show that *ALK* rearrangements are rare but highly actionable targets in LCNEC and that up-front molecular profiling can dramatically impact the disease course of patients with metastatic ALK-driven LCNEC.

## Conclusions

ALK rearrangements may oncogenically drive LCNEC, a rare and clinically aggressive subtype of lung cancer, sensitizing this tumor to treatment with ALK inhibitors. Given the genomic heterogeneity of LCNEC, up-front comprehensive molecular profiling should be offered to patients with advanced LCNEC. Moreover, our study shows that ALK IHC alone is an insufficient diagnostic method to assess the ALK status in neuroendocrine tumors of the lung, requiring integration of FISH and NGS into the diagnostic algorithm.

## Data Availability Statement

Data supporting the findings of this study are available within the article and its supplementary information files or from the corresponding author upon request. In order to minimize the risk of re-identification, individual-level data are not publicly available. However, requests from accredited researchers for access to de-identified individual-level or aggregate data relevant to this manuscript, can be made available upon request by contacting the corresponding author. Accredited researchers should provide contact information, affiliation/organization, and research rationale.

## Ethics Statement

The studies involving human participants were reviewed and approved by Kantonale Ethikkomission, Kanton Zürich, Switzerland (KEK ZH-Nr. 29-2009). The patients/participants provided their written informed consent to participate in this study.

## Author Contributions

DA contributed to data collection, data analysis, and article writing. MH contributed to data collection, data analysis, and article editing. RF contributed to data collection and data analysis. SH contributed to data analysis and article editing. MR contributed to data collection, data analysis, and article editing. JR contributed to data collection, data analysis, and article editing. AC-F contributed to study conception and coordination and article editing. All authors contributed to the article and approved the submitted version.

## Conflict of Interest

SH reports consulting and advisory fees from Bayer outside the submitted work. RF reports grants and consulting and advisory fees from Pierre Fabre, Bayer, Merck, MSD, Astra Zeneca, Novartis, and BMS outside the submitted work. MK reports ownership relationship with Novavax, Biontech, and Mol. Partners outside the submitted work. AC-F reports patent relationship with Astra Zeneca, Bristol Meyer Squibb, Boehringer Ingelheim, Pfizer, Roche, Takeda, MSD, and Janssen-Cilag outside the submitted work.

The remaining authors declare that the research was conducted in the absence of any commercial or financial relationships that could be construed as a potential conflict of interest.

## Publisher’s Note

All claims expressed in this article are solely those of the authors and do not necessarily represent those of their affiliated organizations, or those of the publisher, the editors and the reviewers. Any product that may be evaluated in this article, or claim that may be made by its manufacturer, is not guaranteed or endorsed by the publisher.

## Supplementary Material

Supplementary Figure 1 Hematoxylin-eosin (H-E), synaptophysin (SYN) and Ki-67 IHC stainings of neuroendocrine tumors of the lung. **(A)** H-E, SYN and Ki-67 staining of selected TMA cases comprising typical carcinoid, atypical carcinoid, SCLC and LCNEC. **(B)** H-E, SYN and Ki-67 staining of three *ALK* rearranged LCNEC cases. AC, atypical carcinoid; LCNEC, large cell neuroendocrine carcinoma; SCLC, small cell lung cancer; TC, typical carcinoid; TMA, tissue microarray. Scale bars 200 µm and 2 mm.

Supplementary Figure 2 Schematic diagram illustrating workflow for ALK diagnostics per IHC, FISH and NGS in the retrospective cohort. IHC was assessed first on all evaluable samples (n=365/439). FISH was scored on 28 samples positive by ALK IHC. NGS was performed on 12 samples positive per IHC and with enough available material. ALK, anaplastic lymphoma kinase; IHC, immunohistochemistry, FISH, fluorescence *in situ* hybridization; NGS, next-generation sequencing.

Supplementary Figure 3 Schematic diagram illustrating workflow for ALK diagnostics per IHC, FISH and NGS in 3 index cases. IHC was assessed and was evaluated as positive (3+) on all 3 samples (n=3/3). FISH was scored and resulted positive on 2/2 evaluable samples. NGS confirmed *ALK* rearrangement in all 3 cases. ALK, anaplastic lymphoma kinase; IHC, immunohistochemistry; FISH, fluorescence *in situ* hybridization; NGS, next-generation sequencing
