# Supplementary figures and images for: Targeting ALK in Neuroendocrine Tumors of the Lung
Source: Front Oncol. 2022 Jun 7;12:911294. doi: 10.3389/fonc.2022.911294 (PMC9214311; doi:10.3389/fonc.2022.911294)

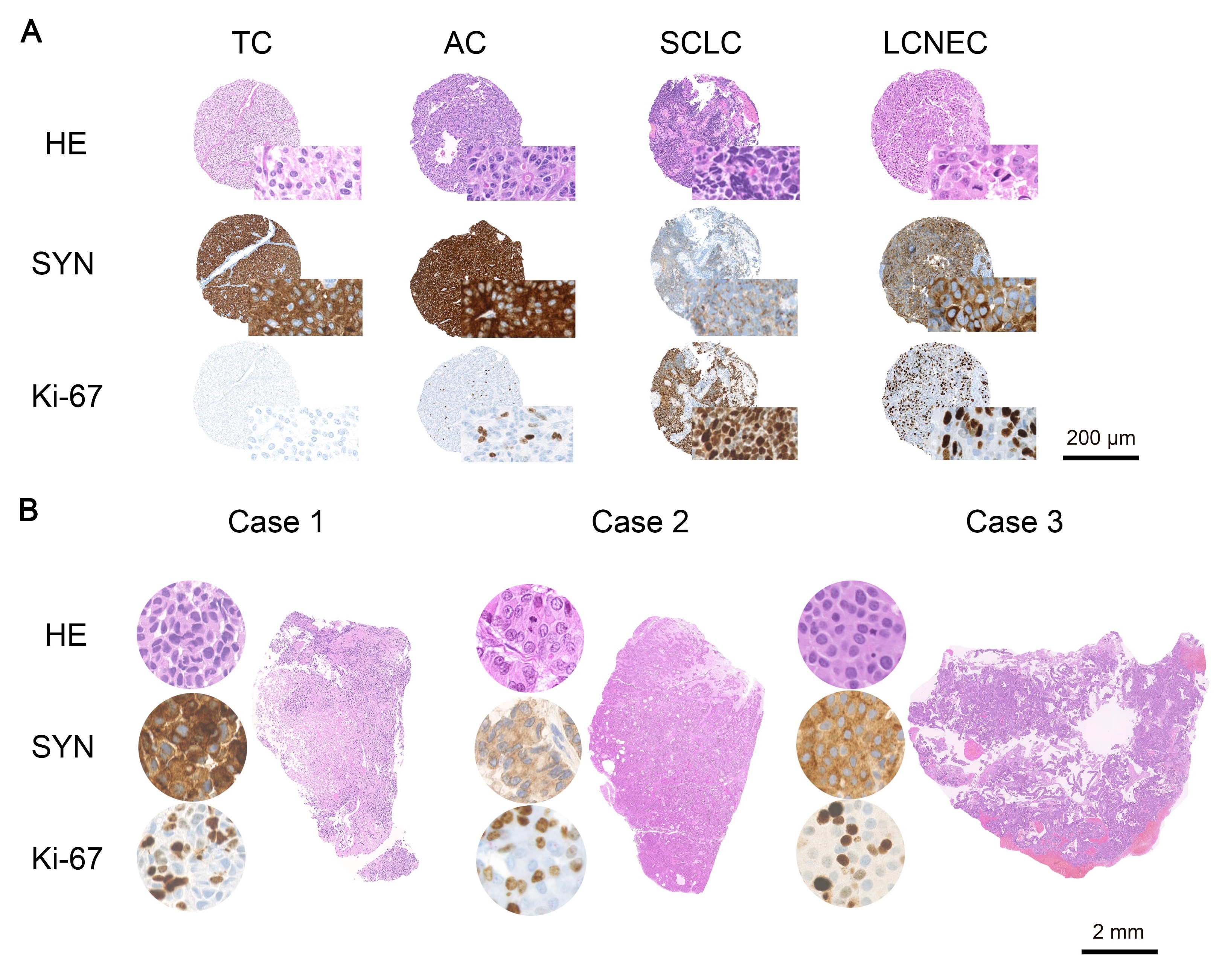

Supplement: Supplementary Figure 1 — Hematoxylin-eosin (H-E), synaptophysin (SYN) and Ki-67 IHC stainings of neuroendocrine tumors of the lung. (A) H-E, SYN and Ki-67 staining of selected TMA cases comprising typical carcinoid, atypical carcinoid, SCLC and LCNEC. (B) H-E, SYN and Ki-67 staining of three ALK rearranged LCNEC cases. AC, atypical carcinoid; LCNEC, large cell neuroendocrine carcinoma; SCLC, small cell lung cancer; TC, typical carcinoid; TMA, tissue microarray. Scale bars 200 µm and 2 mm. [file Image_1.jpeg]

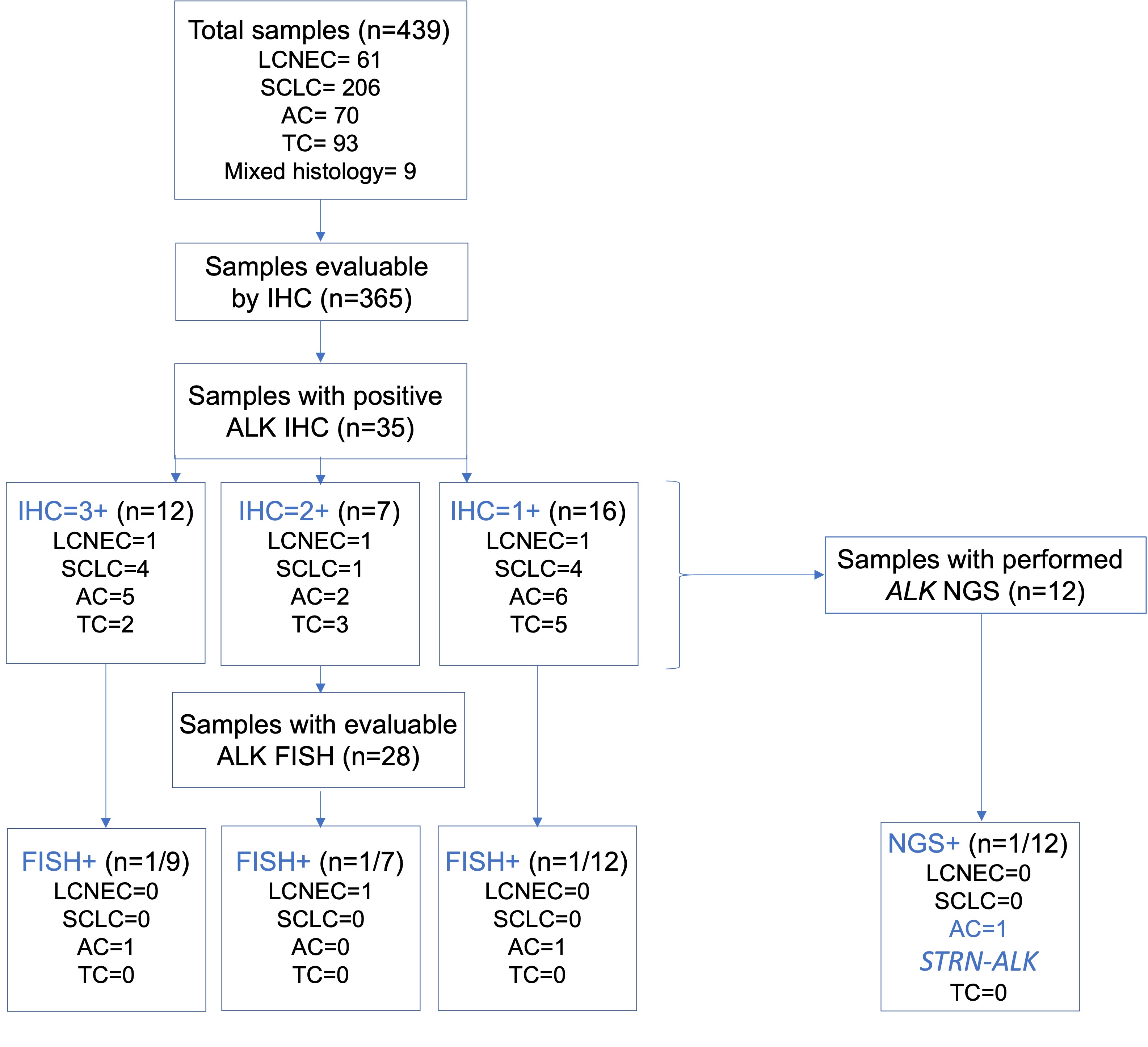

Supplement: Supplementary Figure 2 — Schematic diagram illustrating workflow for ALK diagnostics per IHC, FISH and NGS in the retrospective cohort. IHC was assessed first on all evaluable samples (n=365/439). FISH was scored on 28 samples positive by ALK IHC. NGS was performed on 12 samples positive per IHC and with enough available material. ALK, anaplastic lymphoma kinase; IHC, immunohistochemistry, FISH, fluorescence in situ hybridization; NGS, next-generation sequencing. [file Image_2.jpg]

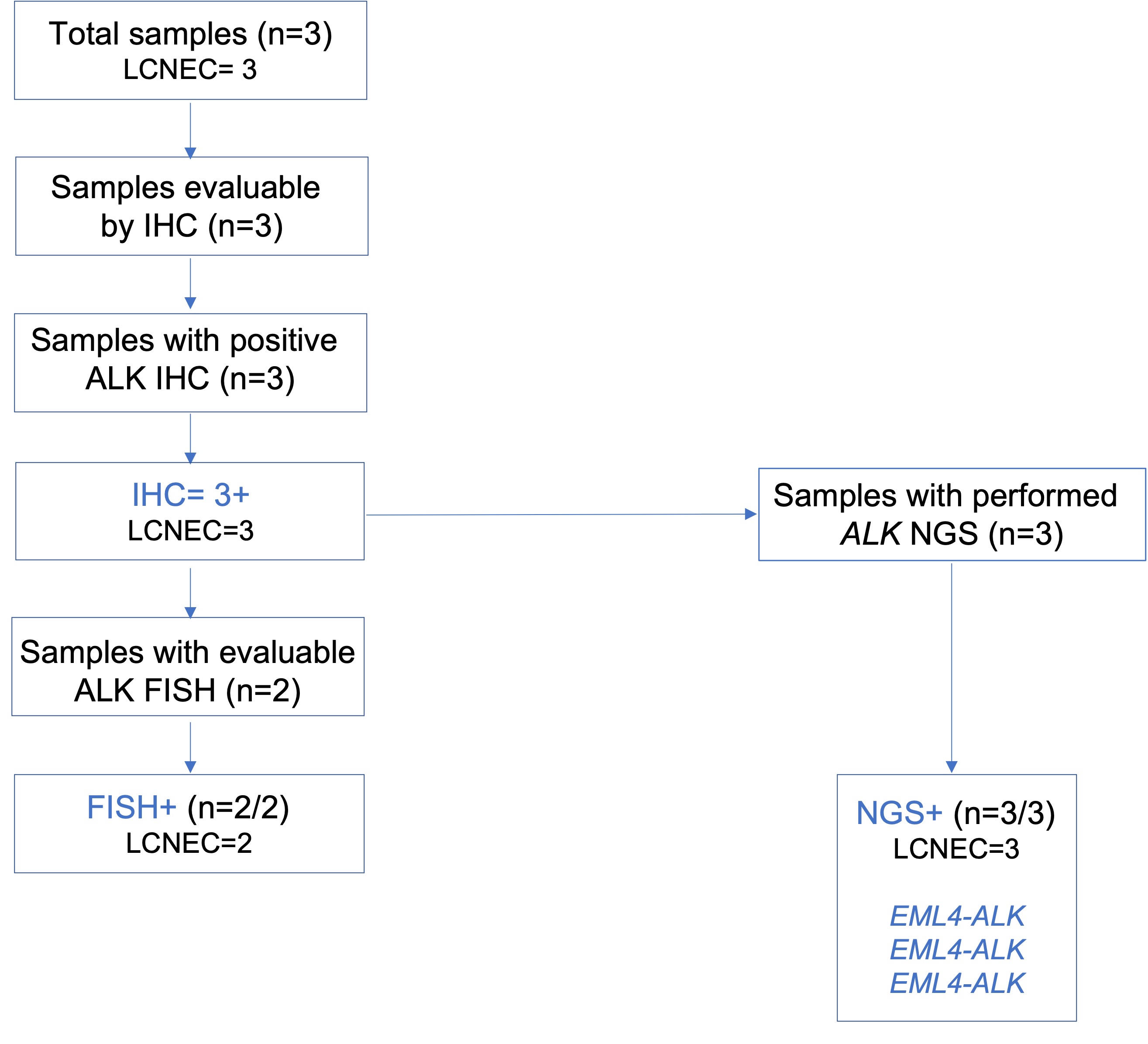

Supplement: Supplementary Figure 3 — Schematic diagram illustrating workflow for ALK diagnostics per IHC, FISH and NGS in 3 index cases. IHC was assessed and was evaluated as positive (3+) on all 3 samples (n=3/3). FISH was scored and resulted positive on 2/2 evaluable samples. NGS confirmed ALK rearrangement in all 3 cases. ALK, anaplastic lymphoma kinase; IHC, immunohistochemistry; FISH, fluorescence in situ hybridization; NGS, next-generation sequencing [file Image_3.jpg]
